# Advances and challenges of operational seasonal prediction in Pacific Island Countries

**DOI:** 10.1038/s41598-022-15345-w

**Published:** 2022-07-06

**Authors:** Yun-Young Lee, WonMoo Kim, Soo-Jin Sohn, Bo Ra Kim, Sunny K. Seuseu

**Affiliations:** 1 APEC Climate Center, Busan, 48058 South Korea; 2 Secretariat of the Pacific Regional Environment Programme, Apia, Samoa; 3 Present Address: Green Technology Center, Seoul, 04554 South Korea; 4 Present Address: Climate Services and Capacity Building Unit, Science Division, UN Environment Programme (UNEP), Nairobi, Kenya

**Keywords:** Climate sciences, Environmental sciences

## Abstract

Seasonal climate forecasts play a critical role in building a climate-resilient society in the Pacific Island Countries (PICs) that are highly exposed to high-impact climate events. To assist the PICs National Meteorological and Hydrological Services in generating reliable national climate outlooks, we developed a hybrid seasonal prediction system, the Pacific Island Countries Advanced Seasonal Outlook (PICASO), which has the strengths of both statistical and dynamical systems. PICASO is based on the APEC Climate Center Multi-Model Ensemble (APCC-MME), tailored to generate station-level rainfall forecasts for 49 stations in 13 countries by applying predictor optimization and the large-scale relationship-based Bayesian regression approaches. Overall, performance is improved and further stabilized temporally and spatially relative to not only APCC-MME but also other existing operational prediction systems in the Pacific. Gaps and challenges in operationalization of the PICASO system and its incorporation into operational climate services in the PICs are discussed.

## Introduction

Seasonal climate forecasts, which predict the tendency of major climate variables such as near surface temperature and precipitation a couple of months to even a year in advance, play a critical role in building a climate-resilient society. Long before the establishment of modern observation networks, weather- and climate-related traditional knowledge^1,2^ served communities to be better prepared for upcoming climate events. With a growing number of observational records and our improved understanding of the climate system, the empirical or statistical seasonal climate forecasts that utilize historical lead-lag relationships have greatly improved our ability to predict seasonal climate^3,4^. Statistical prediction is possible when the climate system is stationary, as some major climate drivers like El Niño and La Niña persist and exert well-organized teleconnection patterns on seasonal timescales^5–7^. As a result, local seasonal climates are relatively easier to predict using statistical methods when the seasonal climate drivers are in an active phase and where the teleconnection is strong. On the other hand, dynamical climate prediction systems, which can be suitable under a changing climate, have made remarkable progress in the past few decades^8–12^. There is skill to predict some climate drivers several seasons in advance^11^, leading to reliable local seasonal forecasts one or two seasons in advance. Moreover, by combining different information from multiple models, model-dependent systematic biases can cancel each other out to produce a less erroneous prediction compared to a single-model prediction. Therefore, Multi-Model Ensemble (MME) prediction systems tend to have higher prediction skill than individual models^13–15^. Currently, MME products are available through the World Meteorological Organization Lead Centre for Long-Range Forecast (WMO LC-LRF)^16^, APEC Climate Center (APCC)^17^, North American MME (NMME)^18^, Copernicus Climate Change Service (C3S), and others. Here we introduce a hybrid approach for the operational climate forecast in the PICs, which utilizes statistically tailored dynamical MME seasonal prediction.

### a. Operational systems in the Pacific: statistical approach

Many of the Pacific Island Countries (PICs) are already utilizing various types of high-quality seasonal prediction information for their operational seasonal outlooks. Among them are purely statistical prediction, predictions from a single dynamical GCM, and MME prediction systems, each with its own benefits and drawbacks. A major source of prediction information is the Seasonal Climate Outlooks in Pacific Island Countries (SCOPIC^19^), which was developed as part of the Climate and Oceans Support Program in the Pacific (COSPPac, http://cosppac.bom.gov.au/) funded by the Australian Department of Foreign Affairs and Trade (DFAT). SCOPIC generates seasonal outlooks for rainfall, temperature, or other climate-related factors using statistical techniques to determine forecast probabilities based on historic data. It was originally designed to ingest various predictors as inputs, but in its actual operation, the seasonally averaged El Niño-Southern Oscillation (ENSO) index (NINO3.4) is utilized for its high impact on the region and to minimize the risk of introducing artificial skills or overfitting. The dominance and the persistence of ENSO provides high prediction skills (averaged LEPS (Linear Error in Probability Space) score around 10–20%) over many PICs, particularly those under the direct influence of well-organized canonical ENSO impacts. However, the technical limitations of using only the canonical ENSO index lowers the prediction skills in regions or seasons where the ENSO signal is weak or non-canonical^20–22^. Also, any statistical approach is heavily based on the stationarity of the climate state, so it may also suffer under a changing climate when either ENSO or its impact changes significantly^23–25^.

### b. Operational systems in the Pacific: dynamical approach

Dynamical prediction systems have provided supplementary information for operational seasonal outlooks in the PICs. The Predictive Ocean Atmosphere Model for Australia (POAMA), a physics-based single dynamical model, served the region for almost 10 years (2013–2020), particularly for regional-scale climate outlooks, and has been replaced by the newly developed ACCESS-S (Australian Community Climate and Earth-System Simulator-Seasonal prediction) system^26^ since 2020. Despite its low resolution, POAMA showed operationally reasonable performance for monsoon and ENSO predictions for the last decade. The new ACCESS-S system has higher resolution, thus better representation of major climate drivers and local characteristics^26–28^. The incorporation of the ACCESS-S system into the PICs national climate outlooks is therefore expected to improve the prediction skill of these outlooks. However, individual models have tendencies to have some systematic biases that stem from their parameterization strategy, resolution, etc. MME prediction systems generally outperform a single model approach in terms of prediction skill, but they are relatively low in spatial resolution, targeting general global features. Therefore, MME seasonal predictions, *e.g.*, APCC MME and WMO LC-LRF MME, are also utilized in the region to grasp regional tendencies and to confirm the national climate outlook. Thus, dynamic model outputs need to undergo post-processing and an intensive reassessment process by climate experts in local National Meteorological and Hydrological Services (NMHSs), which may not always be realistic in operational procedures.

### c. Pacific Island Countries’ Advanced Seasonal Outlook (PICASO): hybrid approach

The Pacific Island Countries Advanced Seasonal Outlook (PICASO) is a hybrid statistical–dynamical seasonal climate prediction system for 49 selected stations in 13 countries (see Fig. 5 and Supplementary Fig. 1). It was developed through the Republic of Korea-Pacific Islands Climate Prediction Services project (ROK-PI CliPS; 2014–2017) funded by the Republic of Korea-Pacific Islands Forum Cooperation Fund (RPCF) to assist the Pacific Island NMHSs in generating high quality climate forecasts^29^. Many PICs have islands smaller than the MME grid representation or small-scale topographic features that affect local climate states, making global- or regional-scale prediction information potentially unsuitable for their operational domestic climate outlooks. To improve the national operational seasonal prediction skills, PICASO statistically downscales the dynamical MME prediction to station-level precipitation forecasts. To get the best performance, PICASO only considers the latest MME seasonal prediction fields with the shortest lead time and we call it “lead time 1-month”. For individual models participating APCC MME, however, the lead times relative to a target season varies depending upon operational schedule (Table 1), and it should be noted that the MME produced by APCC is the result of combining forecasts with slightly different lead times. PICASO adopts a strategy that maximizes the prediction skill and meets the computational (*e.g.*, no heavy computation allows it to meet the operational timetable), network (*e.g.*, online communication minimized for countries with limited network speed), and operational (*e.g.*, adhering to national data guidelines restricting sharing of observational data) requirements to follow the region’s operational needs. In this paper, we present the impacts of the hybrid approach in terms of performance improvement and describe the downscaling procedures of predictor selection and relation-based Bayesian regression that are utilized in the PICASO system. The comparison of PICASO with existing systems provides PIC NMHS with key information in their operations. We also review the lessons learned in applying our scientific research to operations.

**Table 1:** Description of 8 dynamical seasonal climate prediction systems used in PICASO.

Model	Institute	Resolution	Ens	Lead time (months)^†^	Reference
CCSM3^a^	APCC/Korea	T85L26	10	1.2	Jeong et al.^40^
CMCC^b^	CMCC/Itlay	T63L19	9	1	Alessandri et al.^41^
CWB^c^	CWB/Chinese Taipei	T42L18	10	1.5	Liou et al.^42^
CANCM^d^	MSC^e^/Canada	T63L31	20	1	Merryfield et al.^43^
GMAO^f^	NASA^g^/USA	288 × 181L72	11	1.5	Molod et al.^44^
CFSv2^h^	NCEP^i^/USA	T62L64	20	1.2	Saha et al.^45^
PNU^j^	PNU/Korea	T42L18	5	1	Ahn and Kim^46^
POAMA	BoM/Australia	T47L17	33	1	Lim et al.^47^

^a^Community Climate System Model Version 3.

^b^Centro Euro-Mediterraneo sui Cambiamenti Climatici.

^c^Central Weather Bureau of Chinese Taipei.

^d^Canadian Centre for Climate Modeling and Analysis Coupled Climate Model.

^e^Meteorological Service of Canada.

^f^Global Modeling and Assimilation Office.

^g^National Aeronautics and Space Administration.

^h^Coupled Forecast System model version 2.

^i^National Center for Environmental Prediction.

^j^Pusan National University.

^†^An approximate time distance (months) between the date of initial condition and the first day of forecast target season.

## Results

### PICASO tailoring strategy: predictor optimization and relationship-based regression

We first investigate the respective prediction skills, using Heidke Skill Score (HSS) and Linear Error in Probability Space (LEPS) score, of utilizing (a) the expert’s selection of physically meaningful predictors (*i.e.*, optimal predictors) and (b) the relationship-based Bayesian approach. Here, we compare (a) Exp.1—canonical predictor, relationship-based: All predictors are replaced by the model-predicted Niño3.4 index, or (b) Exp.2—optimal predictor, spread-based: The mapping is replaced by a simple linear regression with the predicted variance estimated from the inter-model spread of predictors, with the PICASO methodology (*i.e.*, optimal predictor, relationship-based).

#### Canonical vs optimal predictors

**Figure 1:**
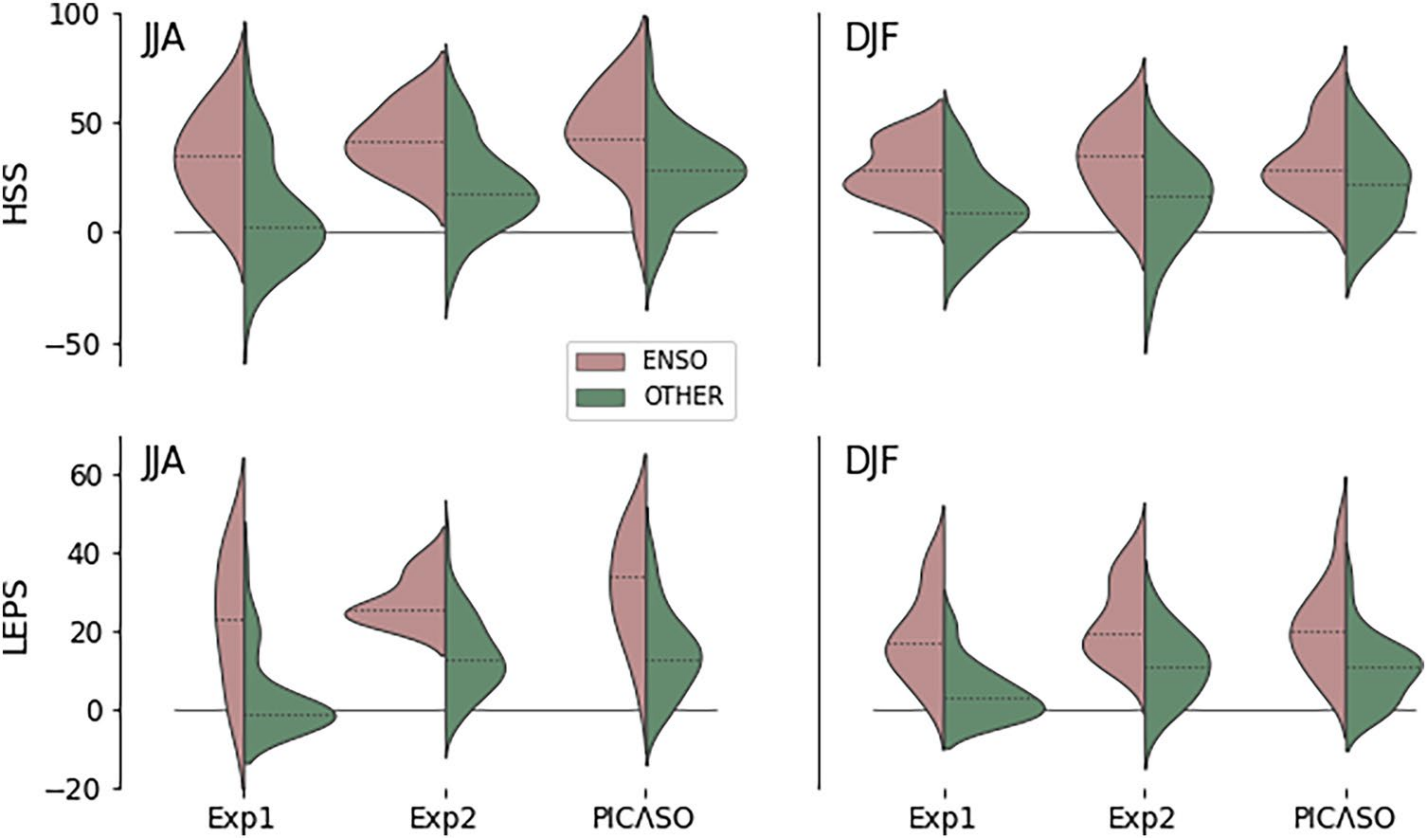
Heidke Skill Scores (HSS; upper) and LEPS scores (lower) for JJA (left) and DJF (right) seasons for the three experiments: (Exp.1) canonical predictor, relationship-based: All predictors are replaced by the model-predicted Niño3.4 index. (Exp.2) optimal predictor, spread-based: The mapping is replaced by a simple linear regression with the predicted variance estimated from the inter-model spread of predictors; and (PICASO) optimal predictor, relationship-based: Both hand-picked optimal predictors and relationship-based Bayesian regression are applied, which are the same methodologies utilized in PICASO. The left and right sides of each violin plot are the distribution of scores for the stations with dominantly impacted by ENSO (denoted as ‘ENSO’; rosy brown), and the stations where other remote forcing dominates or large-scale forcing is obscure (denoted as ‘OTHER’; green), respectively.

Figure 1 indicates that the optimal predictors improved both HSS and LEPS for most seasons over the canonical predictor (*cf.* Exp. 1 *vs* PICASO). The optimal predictors (as in PICASO) are based on the observed dynamic linkage and the reproducibility in DMME (deterministic MME), which are jointly identified by APCC and PIC NMHSs as described in the “Methods” section. On the other hand, the canonical predictor (Exp. 1) is set to the simultaneous MME-simulated Niño3.4 index. It is duly expected that the prediction skills for the stations with limited ENSO impact (*i.e.*, ‘OTHER’ category) should be improved when considering other teleconnections like the Indian Ocean, monsoon, *etc.* compared to only utilizing the Niño3.4 index as a predictor. The improvement of HSS is the most significant in the JJA season (from ~ 0 to ~ 20%). The LEPS scores exhibit stronger effects of introducing the optimal predictors for the ‘OTHER’ category, as shown by significantly reduced linear errors during all seasons (from ~ 0 to ~ 10–20%). Interestingly, however, the improvement of prediction skills for the stations with strong ENSO impact (‘ENSO’ category) is also prominent in many seasons. The HSSs of the optimal predictors (PICASO) tend to be higher than that of the canonical predictor (Exp. 1), except for in the DJF season, and the LEPS scores improved for all seasons. This skill improvement is due to the introduction of predictors that slightly vary for each station and that have internal structures embedded as their weightings. Thus, the optimal predictors capture the internal spatial structures of ENSO, and it is important to consider differently simulated ENSO flavors in MME to improve station-level prediction. Also, it can be inferred from the results that current GCMs participating in APCC DMME have some ability to predict the spatial diversity of ENSO. The detailed information on the site-specific optimal predictor varies station by station and is accessible through the “Guide” tab of the PICASO program.

#### Spread-based vs relationship-based approach

We then consider the effect of the inter-model spread of predictors in a simple spread-based linear regression (Exp. 2) and the relationship-based Bayesian regression (as in PICASO). The former estimates the forecast uncertainty based on the variance calculated from the individual model runs and the ensemble spread. On the other hand, the latter only considers the uncertainty associated with the prediction-observation relationship from the deterministic MME, as in PICASO. Both approaches give mostly comparable skills and show a statistically insignificant difference, with some seasonal characteristics. For example, in the JJA season, the LEPS scores for the ENSO-dominated stations (‘ENSO’ category) are generally higher for the relationship-based Bayesian approach, but the prediction spread is much smaller for the inter-model spread-based estimates for each station. On the other hand, the HSSs for the stations with limited ENSO impact (‘OTHER’ category) generally increase when applying the relationship-based Bayesian approach. In most other seasons, however, the difference is statistically insignificant at 95% confidence level, and the prediction skills in terms of HSS and LEPS are comparable to each other (not shown). In general, the relationship-based Bayesian approach reasonably estimates the prediction probability even without considering intra- and inter-model spread. Thus, PICASO adopted the relationship-based model and neglect small intra- or inter-model spreads, which also ensured computational efficiency to match operational schedules. However, some discrepancies indicate that there is a remaining possibility of variance prediction for some major climate factors, if not for all aspects.

### PICASO performance: relative to the original APCC PMME prediction system

**Figure 2:**
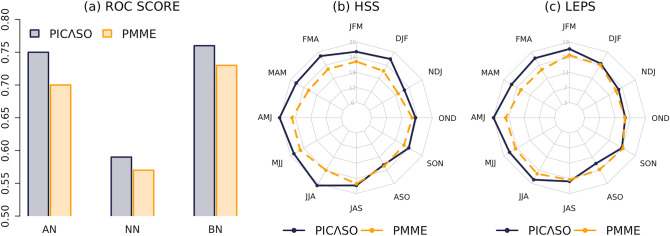
(**a**) Three categories’ ROC scores; and (**b**) HSS and (**c**) LEPS of PICASO and APCC-PMME over 12 seasons for all 49 stations during the training period (1983–2005).

PICASO seasonal forecasts are verified and compared with the APCC PMME precipitation forecasts from the model grid closest to each station during the training period from 1983 to 2005. The Relative Operating Characteristics (ROC) curves show that the Hit Rate (HR) is much larger than the False Alarm Rate (FAR) in PICASO and PMME for all three categories, indicating that both systems are skillful in discriminating events from non-events (not shown). The ROC scores of the above-normal (AN), near-normal (NN), and below-normal (BN) categories are 0.70, 0.57, and 0.73 in PMME and are increased to 0.75, 0.59, and 0.76 in PICASO, respectively (Fig. 2a). Their fractional improvements are 7.1%, 3.5%, and 4.1%, respectively. Based on the official APCC PMME verification derived from the 2019 version reforecast (https://www.apcc21.org/ser/hind.do?lang=en), the regional ROC score differences between the globe and the tropics in PMME are + 0.08, + 0.04, and + 0.12. Compared to this, PICASO achieves meaningful skill improvement (with additional + 0.05, + 0.02, and + 0.03) in the tropics, where the dynamic forecast skill is already much higher than other regions. The symmetrized scores between AN and BN is another key feature of PICASO compared to APCC PMME. The PMME BN score is larger than the AN score, while the two scores become almost similar in PICASO.

Figure 2b,c compares PICASO and APCC PMME skills using HSS and LEPS, and shows their seasonal dependency. The distance from the center is proportional to the skill. The PICASO forecast skill is generally higher than the PMME forecast. The skill improvement, however, varies from season to season. During the transition period from austral summer to winter, the score increase is very robust in both metrics, while there is a slight increase or marginal decrease during the transition period from austral winter to summer in LEPS. Thus, seasonal skill variation is clearer in PICASO in comparison to the APCC PMME. In terms of ENSO seasonal evolution, PICASO significantly outperforms PMME during the ENSO 'decaying' season (from FMA to MJJ), while PICASO does not show dramatic skill improvement in the ENSO 'developing' season (from JAS to OND). Therefore, it is worthwhile to look further into the prediction skill of PICASO in terms of ENSO conditions.

**Figure 3:**
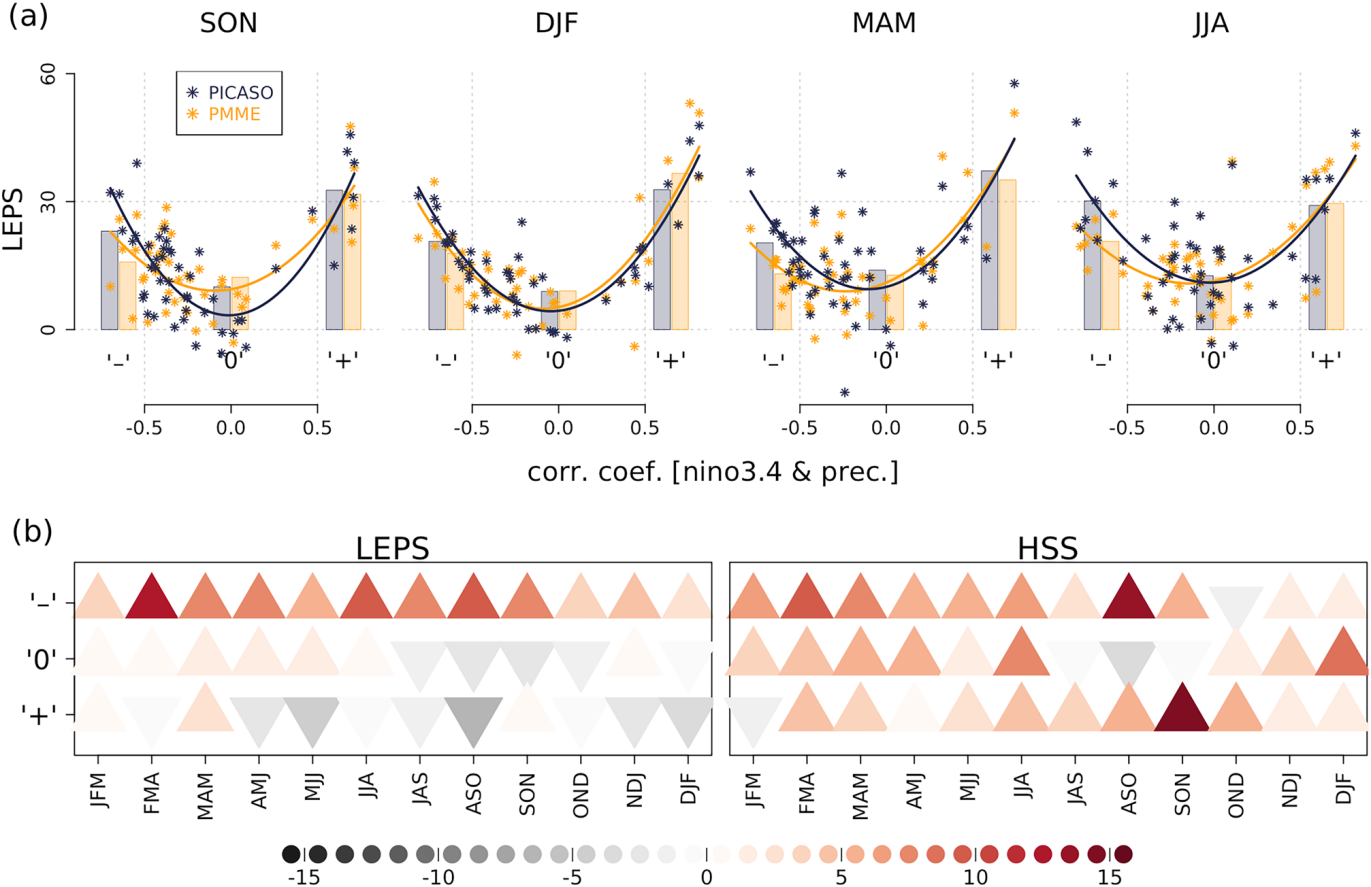
(**a**) Scatter plot of 49 stations between the Niño3.4-precipitation correlation coefficient and LEPS score of (navy) PICASO and (orange) APCC-PMME for four seasons during the training period (1983–2005). The three bars in each panel represent the averaged LEPS of stations for three station categories in terms of their rainfall relationship with ENSO: ‘–’, ‘0’, and ‘+’, respectively. Second-order polynomial line least square fitted to 49 points is delineated for each of the two systems. (**b**) Predictive skill (LEPS and HSS) differences between PICASO and APCC-PMME for three station categories and individual 12 seasons. Reddish upper triangle (grayish lower triangle) indicates the higher (lower) skill of PICASO relative to APCC-PMME.

The dynamical seasonal forecast outperforms when or where the climate system is tightly connected to slowly varying ocean signals^15,30,31^. Consistently, ENSO is the most frequently selected large-scale predictor for the statistical correction in PICASO. Therefore, it is worthwhile to understand how the PICs forecast skill varies regionally, and their reliance on ENSO signals. Based on the rainfall reliance on ENSO, the 49 stations can be grouped into three categories: (1) positive relationship (‘+’), (2) negative relationship (‘–’), and (3) obscure relationship (‘0’) with ENSO. The classification criteria are correlation coefficients of ± 0.5 between three months rainfall and the Niño3.4 index; for each station, if the correlation coefficient is over + 0.5, it is classified as a ‘+’ category, and if the correlation coefficient is less than − 0.5, then it is classified as a ‘−’ category, and if the correlation coefficient is between − 0.5 and + 0.5, then it is classified as ‘0’ category. In APCC-PMME, the LEPS scores for stations with strong ENSO reliance are larger than for stations with no reliance, regardless of season (orange scatters and bars in Fig. 3a). The score is quite high for the positively impacted stations (‘+’) that are located over the southern central Pacific where ENSO-related South Pacific Convergence Zone (SPCZ) variation is dominant (Supplementary Fig. 2). For the negatively related stations (‘–’), the scores for both austral spring and autumn are low. The stations with obscure relationship (‘0’) show the worst PMME scores when heading to austral summer. Notice the asymmetric scores between positively and negatively related stations for PMME: the LEPS score is much lower for the negative ones than that of the positive ones, and is similar to that of the obscure ones during MAM.

After tailoring, the LEPS score increases for negatively related stations for all seasons. Thus the 49 stations’ skill distribution becomes more symmetric (navy scatters and bars in Fig. 3a). During JJA, LEPS of negatively related stations is higher than the positive ones, while it is still asymmetric in other seasons. The skill improvement of PICASO is most dominant for the stations where rainfall exhibits a negative relationship to ENSO, but PICASO rarely outperforms PMME for the stations with a positive or obscure relationship with ENSO.

Figure 3b displays the skill difference between PICASO and the PMME in terms of ENSO reliance by the seasons. As mentioned in Fig. 3, the LEPS score of ‘–’ increases in all 12 seasons, particularly for FMA, JJA, and ASO (Fig. 3b). For ‘0’, the LEPS score marginally increases from JFM to JJA. For ‘+’, the LEPS score tends to decrease in all seasons except for JFM, MAM, and SON. Unlike the LEPS score, HSS shows skill improvement in most cases and the amount of score increase is not very different among the three station categories. For ‘–’, HSS seasonal variation shows a similar tendency to that of the LEPS score, but the maximum increase is found in ASO, not in FMA. For ‘0’, HSS mostly increases, except in three seasons from JAS to SON. For ‘+’, HSS increase is observed in all seasons except JFM, which is very different from the LEPS score. This suggests that performance comparison between the two systems can be different, dependent on which verification metric is utilized. As mentioned in Appendix B, LEPS measures the difference in probabilistic distribution between forecast and observation, while HSS measures the deterministic category of maximum probability. When a forecast shows multiple categories with similar probabilities, HSS and LEPS can tell different stories, like in the case of ‘+’.

### PICASO performance: relative to existing operational prediction systems in PICs

**Figure 4:**
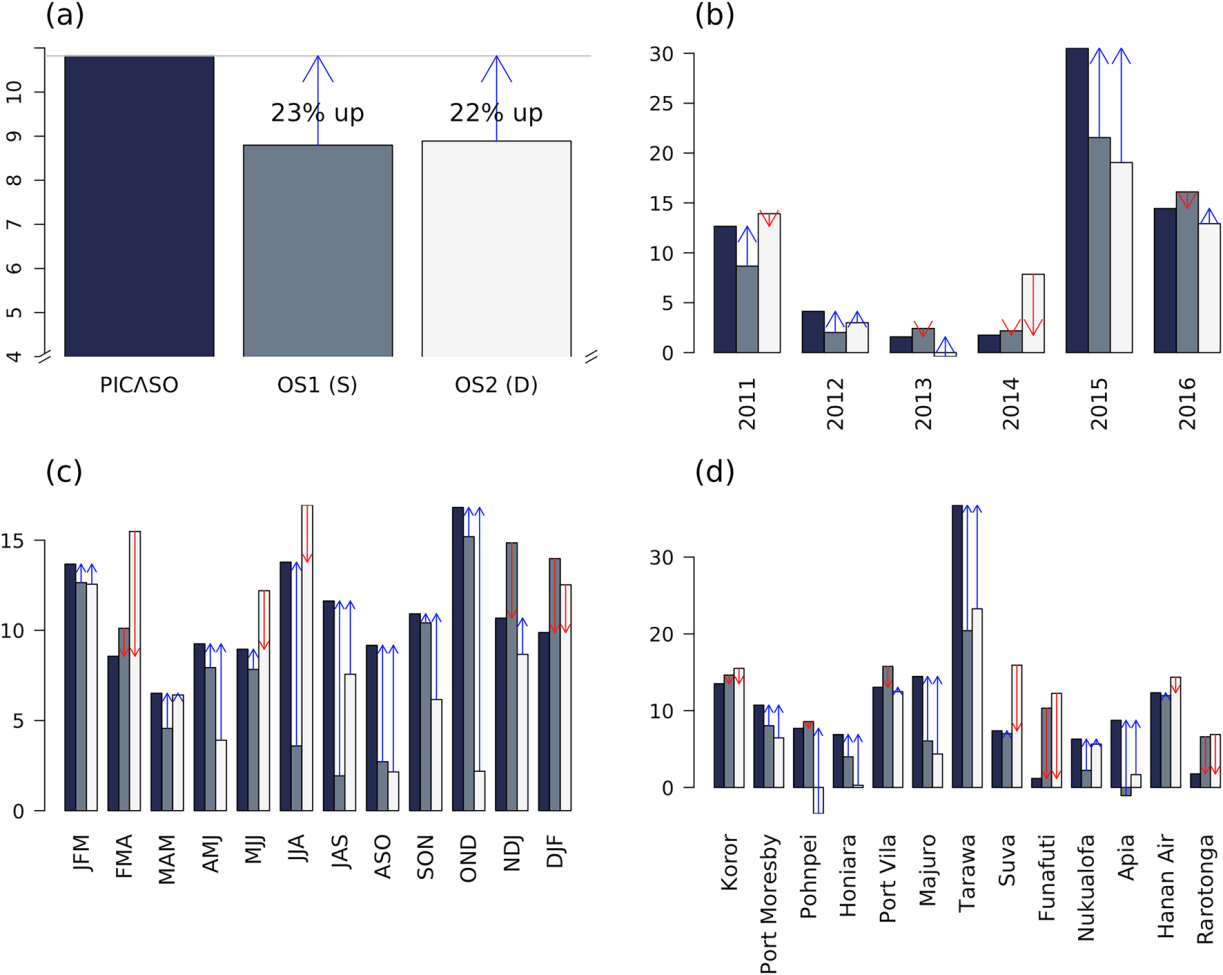
(**a**) LEPS scores of PICASO and two existing operational systems, OS1 (statistical model) and OS2 (dynamic model) in the Pacific derived from the stations located in the capital of 13 PICs for 12 seasons during six validation years (2011–2016) and their variation with the individual (**b**) 6 years, (**c**) 12 seasons, and (**d**) 13 stations. Blue (red) arrows indicate the score increment (decrement) of PICASO from each of the other two systems.

The PICs have issued their official national and regional seasonal forecasts utilizing the Online Climate Outlook Forum (OCOF) resource since October 2007 (see https://www.pacificmet.net/products-and-services/online-climate-outlook-forum). For the period of analysis, the primary sources of prediction information utilized in OCOF are from one statistical (OS1) and one dynamical (OS2) operational system (see the “Introduction” section a and b) developed by the Australian Bureau of Meteorology (BoM). In this section, we compare the official forecast verification (LEPS) information from the existing operational prediction systems collected from OCOF (for 13 PICs capital stations), to PICASO. The overall LEPS score of PICASO is approximately more than 20% higher relative to the other two systems (Fig. 4a).

Detailed LEPS score diagnostics of 13 PIC stations during six forecast years suggest that prediction performance varies yearly and seasonally (Fig. 4b,c). All three systems show higher scores during prolonged strong El Niño years (2015–2016) and a moderate La Niña year (2011), compared to other years with no significant ENSO signal, implying strong ENSO phase dependency for the PICs rainfall prediction. In terms of seasonal skill variation, there is a noticeable difference between statistical and dynamical models. In the dynamical model, the seasonal skill barrier is evident during the ENSO transition season. However, in the statistical model, the lagged skill barrier is observed during the austral fall to winter season, whose initial condition rarely has active ENSO forcing. PICASO, a hybrid system, exhibits a combined feature of the two systems, showing not only high skill during austral summer as indicated with dynamical systems, but also considerable skill improvement during transition periods, particularly from the austral winter to summer by adopting the benefits of both the dynamical and statistical methods.

This study further analyzes how forecast skill varies regionally among the three systems, as none of the systems exhibits regionally uniform performance (Fig. 4d). All systems commonly show the highest score at Tarawa, Kiribati, which is located in the equatorial Pacific. However, the stations with the lowest skills are quite different among the three systems: Funafuti for PICASO, and Apia and Pohnpei for the statistical and dynamical systems, with occasional negative skill scores. The LEPS score comparison among the three systems reveals that PICASO outperforms the other systems in almost half of the stations (Port Moresby, Honiara, Majuro, Tarawa, Nukualofa, and Apia), suggesting the skill improvement is most evident within the warm-pool region or near the marginal area. However, in Funafuti and Rarotonga, PICASO skill is lower than both other systems, implying that further research is required to revise the predictors and/or tailoring algorithm in PICASO for those stations.

**Figure 5:**
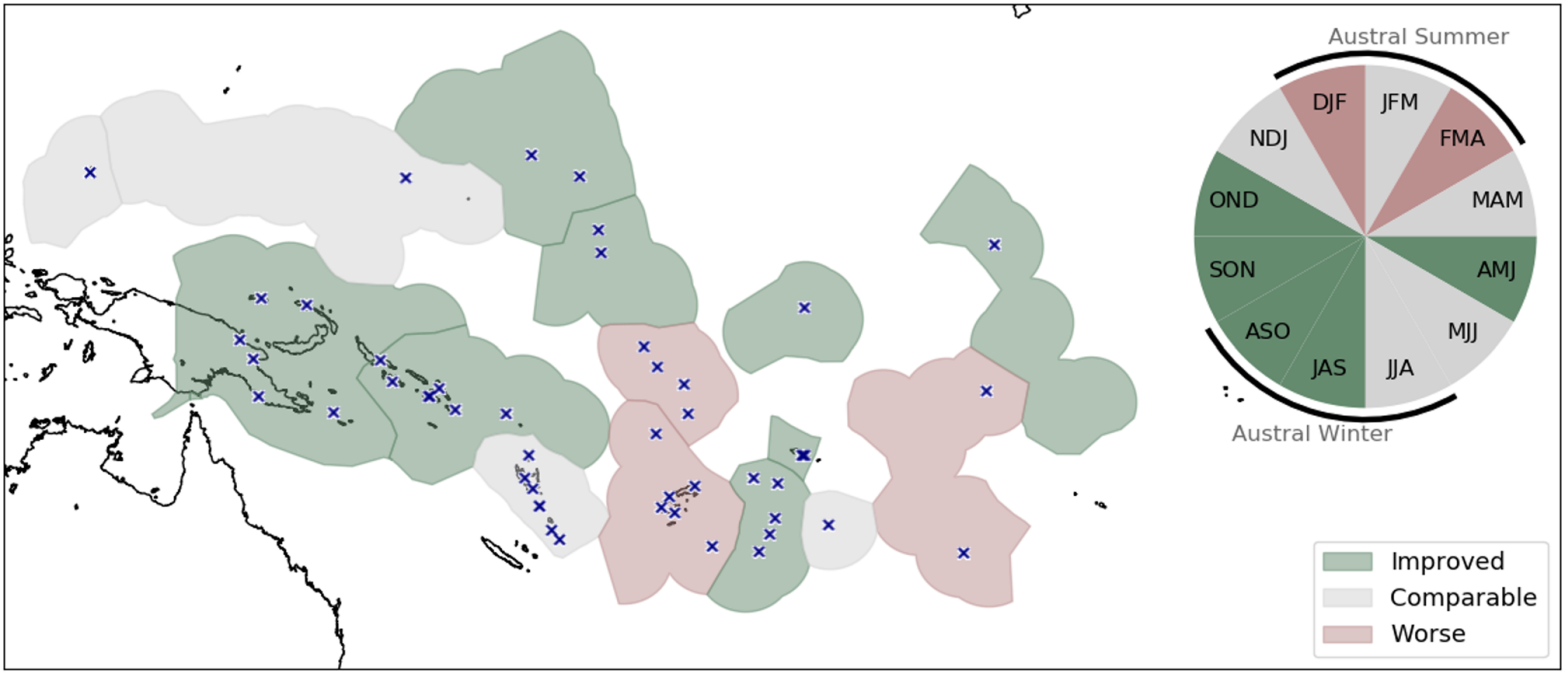
(Map) spatial distribution and (pie) temporal variation of relative PICASO performance inferred from 13 capital stations’ LEPS scores. Dark green, light gray, and rosy brown colors indicate the regions where the PICASO skill is improved, comparable to, or worse than the existing operational systems, respectively. The locations of 49 stations analyzed in this study are marked with “x”.

It would be beneficial to the operational forecast service to compare the regional/seasonal performance in general. Figure 5 displays the spatio-temporal summary of the LEPS score. PICASO performs better than the other systems over the off-equatorial region in the western Pacific and over the central to eastern tropical Pacific; and shows comparable skills over the northwestern and southwestern Pacific, as well as over the southern central Pacific. However, PICASO performance is relatively poor over the southern central Pacific, where rainfall is strongly influenced by the SPCZ. This may be because the seasonal and meridional variation of SPCZ is not well simulated in dynamical prediction systems^32^. In terms of seasonal variation, PICASO functions especially well from the austral autumn to winter compared to other seasons. Gross skill improvement, accompanied by regional and seasonal details, can provide the rationale for utilizing the PICASO system in operations by the PICs NMHS staff to generate better seasonal forecasts. In addition, this information can help develop a strategy to incorporate a newly developed system into operation, and further suggest the importance of diversifying prediction resources and building consensus in the Pacific region.

## Discussion

The dynamical-statistical hybrid system, PICASO, was developed to enhance the seasonal prediction capacity of the PICs. This system provides the PICs NMHS with direct access to APCC’s DMME products by tailoring large-scale predictions into station-level national forecasts. The tailoring strategy leads to a significant skill improvement for all seasons regardless of their relevance to ENSO, and the relationship-based approach effectively covers the forecasted probability distribution. General skill improvements are achieved by considering physical modes of the climate system, such as the state of ENSO, activities of the Indian Ocean, Inter-Tropical and South Pacific Convergence Zones, and monsoon, their reproducibility in the DMME system, and their linkage to station-level climate phenomena. The observation–prediction relationship-based Bayesian regression estimates the probability of tailored seasonal prediction of station-level precipitation at a reasonable level, while avoiding time-intensive calculation of intra- and inter-model spread in the predictor variables. However, there are some interesting aspects that need further attention. First, aside from the expected skill improvement in the ‘OTHER’ category, the improvement in the ‘ENSO’ category through predictor optimization suggests that ENSO diversity is an important factor for better seasonal prediction in the PICs, and that GCMs capture some sub-structures. Second, although considering only the observation–prediction uncertainty is sufficient in estimating the forecast spread in Bayesian regression, the superior skill of inter-model-spread based variance estimation (*e.g.*, Exp. 2 of JJA ‘ENSO’ case) reveals a possibility of variance prediction using the intra- and inter-model spread.

By utilizing physically meaningful predictors together with a relationship-based Bayesian regression, PICASO is shown to be outperforming the APCC PMME in three different verification metrics. In PICASO, ‘events’ and ‘non-events’ are well discriminated compared to PMME, particularly for the AN category. In addition, the deterministic category and probability distribution agreements are considerably increased during the austral spring and summer. Statistical tailoring, however, performs differently in different stations. Stations with rainfall strongly connected to tropical eastern Pacific Ocean cooling (La Niña) indicate robust skill increase, while other stations may not. Current GCMs do not fully simulate the asymmetric response to ENSO and the symmetric response in the GCM is mainly reflecting the positive phase (El Niño), whose amplitude is much larger than the negative phase (La Niña). Therefore, far distant off-phased response over the warm-pool region should have large temporal and spatial biases in the relationship with ENSO. In PICASO, statistical correction improves forecasts for stations located outside of the major ocean warming/cooling area of ENSO by utilizing the systematic relationship between observed rainfall and simulated large-scale pattern of climate drivers and variables outside the Pacific. Ultimately, the spatial and temporal skills have been stabilized in PICASO.

Despite the performance improvement of PICASO, introducing a new system into existing operational forecasts in PIC NMHSs has been and will continue to be a challenge. Although there were high levels of positive feedback from capacity-building programs and visibility events around the release and performance of PICASO, actual operational use has not been widespread in the PICs. There may be a few different factors that contribute to this gap between enthusiasm over the new system and actual operational use: (1) technical limitations of PICASO, (2) absence or inflexibility of Standard Operating Procedures (SOPs), and (3) staffing constraints.

The main technical limitations of PICASO are its inability to include stations that are new or have missing data due to its statistical optimization approach, as well as a later monthly outlook release date due to its predictions being based on MME. In terms of SOPs, many PIC NMHSs have yet to establish official SOPs for generating national climate outlooks. This leads to NMHS officers utilizing forecast systems that are already familiar, making it less likely for them to introduce a new prediction system despite its technical advantages. Regarding capacity, PIC NMHSs are often constrained by a lack of human and financial resources to deliver high-quality climate services, utilizing different forecast and verification methodologies^33^. Most NMHS in the region operate with poor infrastructure and limited capacity, mainly relying on external support to provide basic climatological services^34^. Thus, there may be a lack of full understanding or awareness of the benefits of PICASO's increase in performance.

There is intention and hope for the Republic of Korea-Pacific Islands Climate Prediction Service (ROK-PI CliPS) Phase 2 project and the Green Climate Fund (GCF) funded United Nations Environmental Programme (UNEP) project “Enhancing climate information and knowledge services for resilience in 5 island countries of the Pacific Ocean (FP147; https://www.greenclimate.fund/project/fp147)” to address some of the above factors to increase the operational use of PICASO in the Pacific Islands. SPREP’s Online Climate Outlook Forum (OCOF) and the newly opened Pacific Climate Change Centre (PCCC) e-learning platform are both effective modes of engagement in the PICs. Through this type of regular online support, participants increase their capacity and knowledge around various forecast and prediction methodologies, and increase their abilities to comprehensively utilize various prediction systems when generating outlooks.

Lastly, although PICASO indicates comparable or sometimes enhanced skills when compared to other existing seasonal prediction systems in the PICs, each system has its strengths and weaknesses based on its approach. By comparing and utilizing different information from an observation-based statistical approach, single dynamical model prediction, and hybrid MME-based PICASO, the quality of the national climate outlooks can be further improved.

## Methods

### Model and observation data

PICASO is based on the APCC deterministic MME (DMME) prediction, although APCC issues probabilistic MME (PMME)^35^ as its official product (https://apcc21.org). Station-level precipitation forecast in PICASO is conducted by making estimates from relevant large-scale patterns from the APCC DMME which is a simple arithmetic composite of bias-corrected anomalies predicted by 8 dynamical prediction systems. Table 1 briefly describes the subset of climate models in the APCC DMME utilized in PICASO. The reference period for climatology calculations and PICASO development is 1983 to 2005. The APCC PMME seasonal climate prediction is the composite of uncalibrated parametric Gaussian fitted model probability, with model weights inversely proportional to the errors in forecast probability associated with the model sampling errors. The final PICASO results are compared with the APCC PMME seasonal climate prediction.

Cooperation between SPREP and PIC NMHSs allowed access to station-scale observed precipitation data from 49 stations in 13 PICs, with the understanding that the data would only be utilized for research and development purposes related to PICASO. These data points are seasonally (3-month) averaged station rainfall measurements monitored by respective NMHSs, from stations sparsely distributed over the expansive Pacific Ocean area. The verification scores of the existing systems are extracted from the operational regional climate outlooks. The skill comparison is limited to the stations located in the capital of each country that are available through regional climate outlooks.

### Pacific Island Countries Advanced Seasonal Outlook (PICASO) methodology

PICASO is a hybrid statistical–dynamical seasonal climate prediction system based on APCC DMME. Climate experts from both APCC (expertise in APCC MME products) and the PICs NMHSs (expertise in local climate system) extracted large-scale patterns of climate variables that are associated with observed rainfall. The optimal predictors are then identified based on the discussions among APCC and PICs NMHSs experts (see also Sohn et al. (2018)). We then performed probabilistic Bayesian regressions onto the historical rainfall observation for each station to generate station-level prediction information.

### Linking large-scale prediction and station rainfall

PICASO produces a statistically downscaled precipitation prediction at each station by utilizing APCC DMME’s large-scale pattern as a predictor. The station-level precipitation is mainly governed by large-scale circulation, as well as more complicated local effects, *e.g.*, orographic features, inherent nonlinearity, and physical processes. Current GCMs reasonably simulate the large-scale field, allowing tailoring from large-scale climate information to station-level rainfall^36–38^. In this study, the large-scale pattern is selected based on two criteria, *i.e.*, the observed dynamic linkage and the reproducibility in DMME. The former provides a physical basis for the choice of predictor, and the latter provides applicability of DMME prediction in linking the large-scale to station rainfall. To assess the large-scale pattern that impacts the local rainfall variability, we conducted a correlation analysis between precipitation at each station and large-scale circulation variables for both observations and DMME prediction for each season. The candidate variables for large-scale DMME prediction are sea surface temperature (SST), sea level pressure, zonal and meridional wind at 850 hPa, and precipitation. In order to avoid the artificial selection of large-scale fields, we considered the cross-validated correlation map between station rainfall and large-scale variables in screening the predictor domain. To avoid unexplained uncertainty in the intra- and inter-model spread, the DMME field is utilized for the large-scale predictor selection instead of directly using individual multi-model outputs.

We selected one variable and the corresponding domain with correlation coefficients serving as the weighting function by considering the statistical significance of the correlation pattern, its reproducibility by MME, the representability of well-known predictability source, and physical consistency in correlation patterns among variables. The selected predictor variable and its domain consequently indicates a climate driver or large-scale circulation pattern, which may affect station-level rainfall during the targeted season. It is then multiplied to the DMME to estimate the predictor value for each station and each season. Approximately 83% of 588 cases (49 stations and 12 seasons) show that station rainfall is associated with some large-scale pattern, but 17% do not show any related pattern. The latter may be because the rainfall mechanism at those stations during the target seasons is locally complicated and unable to be explained by a predictable large-scale climate variability. Around 52% of the selected optimal predictors are precipitation, followed by 27% being SST. Regardless of variables, 50% of the valid optimal predictors are dominantly associated with ENSO (referred to as ‘ENSO’ category). More specifically, 16% and 34% tend to receive more rainfall during El Niño and La Niña, respectively. Around 50% of the selected optimal predictors are not directly related to ENSO (referred to as ‘OTHER’ category). This implies the usefulness of PICASO and the further benefits of dynamical seasonal prediction when the empirical association between the local rainfall and ENSO is neither clear nor robust (see Figure 2 of Sohn et al.^29^ for more details).

### Generating probabilistic seasonal forecasts from a noisy relationship

PICASO utilizes a Bayesian approach to consider uncertainty associated with the noisy relationship between the predictor and the actual observation. To regress climate model-based predictors onto the observed seasonal rainfall amount, we first assume that the seasonally accumulated rainfall, *y*_*i*_, at the given station is a *t*-distributed random value around the central tendency *μ*_*i*_, scale parameter *σ*, and the normality parameter *ν*:

$${y}_{i} \sim t\left({\mu }_{i},\sigma ,\nu \right)$$

where the central tendency *μ*_*i*_ is estimated from simple regression of the predictor values onto the observational records with the slope *β*_*1*_ and intercept *β*_*0*_, which are both given broad normal priors. The scale parameter *σ* is given a noncommittal uniform prior between lower and upper bounds, and the normality parameter *ν* is given a broad exponential prior.

$${\mu }_{i}={\beta }_{0}+{\beta }_{1}{x}_{i}$$

$$\sigma \sim unif\left(L,H\right)$$

$$\nu -1 \sim \mathrm{exp}\left(K\right)$$

where *β*_*k*_ ~ *N*(*m*_*k*_,*s*_*k*_). As there is no prior knowledge of DMME-based prediction performance at station levels, we assume arbitrarily large standard deviation *s* about the zero-mean *m*. Then, using the 23-year DMME-based predictors and observational records we estimate the regression coefficients and shape parameters as follows:

$$p\left({\beta }_{0},{\beta }_{1},\sigma ,\nu |D\right)=\frac{p\left(D|{\beta }_{0},{\beta }_{1},\sigma ,\nu \right) p\left({\beta }_{0},{\beta }_{1},\sigma ,\nu \right)}{\iint \iint {d\beta }_{0} d{\beta }_{1} d\sigma d\nu p\left(D|{\beta }_{0},{\beta }_{1},\sigma ,\nu \right) p\left({\beta }_{0},{\beta }_{1},\sigma ,\nu \right)}$$

In practice, we compute the posterior distribution of chain length 10,000, 3 times, discarding every first 1000 warming-up sequence to ensure stability using a Bayesian Markov Chain Monte Carlo (MCMC) algorithm. Finally, the forecasts are generated by inserting the predicted central tendency (*μ*_*i*_ = *β*_*0*_ + *β*_*1*_·*x*_*i*_) and the pre-calculated parameters (*σ* and *ν*) into the distribution and collecting all possible results to generate a final posterior distribution.

### Verification metrics

PICASO is designed to provide verification information with two metrics: Heidke Skill Score (HSS) and Linear Error in Probability Space (LEPS) score. HSS measures the fractional skill improvement over the standard forecast in a “categorical” sense. The standard forecast skill represents the fraction of the correct category forecasts that would be expected by chance. For example, in the case of tercile-based forecasts, it is around one third with a sufficiently large sample.

$$\begin{array}{c}HSS= \frac{{SCORE}_{forecast}-{SCORE}_{by\;chance}}{{SCORE}_{perfect\;forecast}-{SCORE}_{by\;chance}}\times 100\end{array}$$

A perfect forecast should have a score of 100, while a perfectly incorrect forecast should have a score of − 50. A zero score is the reference to measure whether a system has a skill or not, relative to a reference random forecast.

Unlike HSS, LEPS places more emphasis on verifying the “probability” rather than deterministic “category” of maximum probability. It quantifies the distance between the position of the forecast and the corresponding observation in their respective cumulative probability distributions as shown below^39^.

$$\begin{array}{c}LEPS= \left[3\cdot \left(1-\left|{P}_{f}-{P}_{v}\right|+{{P}_{f}}^{2}-{P}_{f}+{{P}_{v}}^{2}-{P}_{v}\right)-1\right]\times 100\end{array}$$

*P*_*f*_ is the cumulative distribution function (CDF) of the forecast and *P*_*v*_ is the CDF of the observation. The LEPS ranges from − 100 to 100, and a positive higher score represents a better prediction.

Apart from the above two metrics, the Relative Operating Characteristics (ROC) metric is separately utilized to measure the ability of PICASO to discriminate ‘events’ and ‘non-events’. ROC measures the ratio of Hit Rate (HR) to False Alarm Rate (FAR) with respect to different thresholds of regular sequence, where HR is the fraction of hits of all ‘events’ and FAR is the fraction of false alarms of all ‘non-events’. The ROC score is defined as the area under the ROC curve, delineating HR/FAR values for regular consecutive thresholds. The score ranges from 0 (no hit) to 1 (all hits, a perfect forecast). The diagonal curve where HR equals FAR represents no skill (ROC = 0.5) over a random forecast. Therefore, good forecast systems should have higher HR than FAR, or in other words, the ROC should be much larger than 0.5.

## Supplementary Information

Supplementary Legends.

Supplementary Figure 1.

Supplementary Figure 2.

## Data Availability

APCC-MME seasonal forecast data, both probabilistic and deterministic, are accessible through APCC’s homepage (https://apcc21.org) or its CLimate Information toolKit (CLIK; https://cliks.apcc21.org). The PICASO forecasts can be generated using the software that is downloadable from SPREP, http://clikp.sprep.org/, under conditions. Station-based rainfall measurements are available from the individual NMHS of each respective country with data agreement. The performances of the existing operational systems are gathered from OCOFs (https://www.pacificmet.net/products-and-services/online-climate-outlook-forum).
