# Supplementary material for: Advances and challenges of operational seasonal prediction in Pacific Island Countries
Source: Sci Rep. 2022 Jul 6;12:11405. doi: 10.1038/s41598-022-15345-w (PMC9259583; doi:10.1038/s41598-022-15345-w)
Supplement: Supplementary file 1 — Supplementary Legends. [file 41598_2022_15345_MOESM1_ESM.pdf]

List of supplementary figures

Supplementary Figure 1. (a) a list of 49 stations selected as seasonal prediction target points in PICASO system and (b) maps showing their geographical location (red circles) for individual 13 countries. The figure map was generated by Cartopy (v0.20, Met Office. UK., http://scitools.org.uk/cartopy).

Supplementary Figure 2. Geographical location of stations having ('+', red circles), ('0', grey circles), and ('-', blue circles) relationship for four seasons. The figure map was generated by Cartopy (v0.20, Met Office. UK., http://scitools.org.uk/cartopy).
