# Supplementary figures and images for: Advances and challenges of operational seasonal prediction in Pacific Island Countries
Source: Sci Rep. 2022 Jul 6;12:11405. doi: 10.1038/s41598-022-15345-w (PMC9259583; doi:10.1038/s41598-022-15345-w)

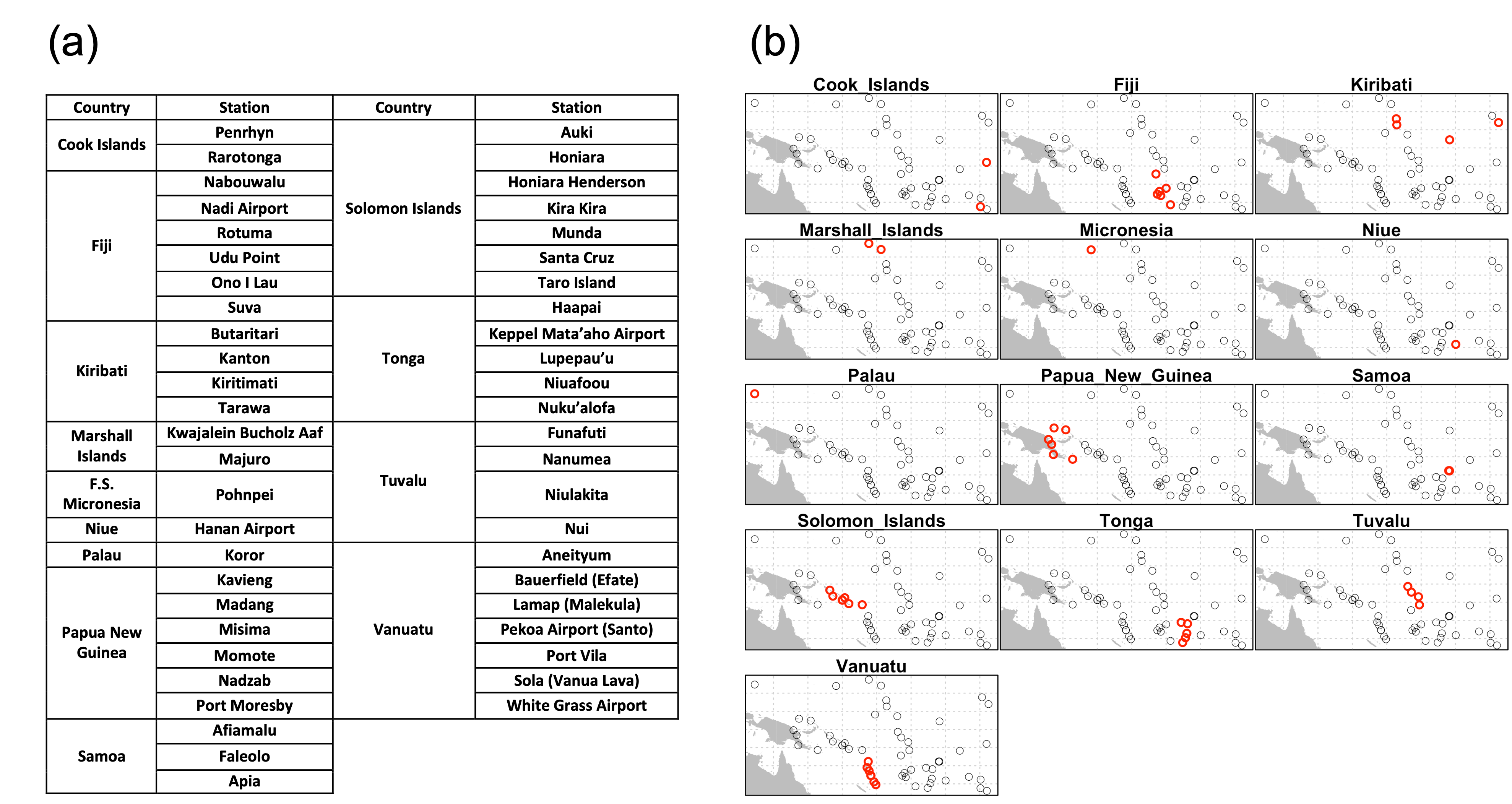

Supplement: Supplementary file 2 — Supplementary Figure 1. [file 41598_2022_15345_MOESM2_ESM.png]

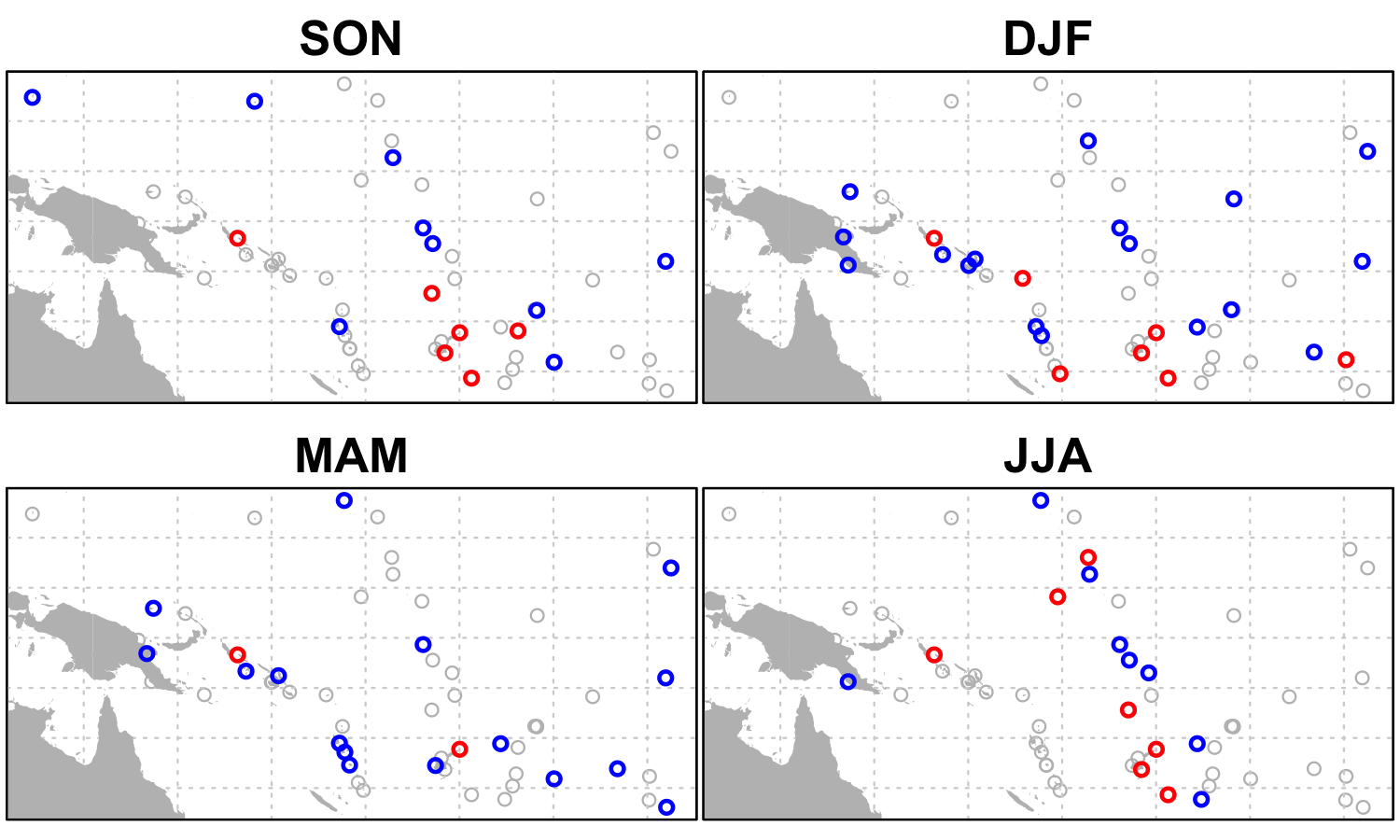

Supplement: Supplementary file 3 — Supplementary Figure 2. [file 41598_2022_15345_MOESM3_ESM.png]
